# The value of kinetic glomerular filtration rate estimation on medication dosing in acute kidney injury

**DOI:** 10.1371/journal.pone.0225601

**Published:** 2019-11-26

**Authors:** Yuenting D. Kwong, Sheldon Chen, Rima Bouajram, Fanny Li, Michael A. Matthay, Kala M. Mehta, David V. Glidden, Kathleen D. Liu

**Affiliations:** 1 Department of Medicine, Division of Nephrology, University of California at San Francisco School of Medicine, San Francisco, CA, United States of America; 2 Department of Medicine, Division of Nephrology, MD Anderson, Houston, TX, United States of America; 3 Department of Pharmaceutical Services, University of California at San Francisco Medical Center, San Francisco, CA, United States of America; 4 Department of Medicine, Division of Pulmonary, Critical Care, Allergy and Sleep Medicine, University of California San Francisco School of Medicine, San Francisco, CA, United States of America; 5 Department of Epidemiology and Biostatistics, University of California at San Francisco School of Medicine, San Francisco, CA, United States of America; 6 Department of Anesthesia, Division of Critical Care Medicine, University of California at San Francisco School of Medicine, San Francisco, CA, United States of America; University of Colorado Denver School of Medicine, UNITED STATES

## Abstract

**Background:**

In acute kidney injury (AKI), medication dosing based on Cockcroft-Gault creatinine clearance (CrCl) or Chronic Kidney Disease Epidemiology Collaboration (CKD-EPI) estimated glomerular filtration rates (eGFR) are not valid when serum creatinine (SCr) is not in steady state. The aim of this study was to determine the impact of a kinetic estimating equation that incorporates fluctuations in SCrs on drug dosing in critically ill patients.

**Methods:**

We used data from participants enrolled in the NIH Acute Respiratory Distress Syndrome Network Fluid and Catheters Treatment Trial to simulate drug dosing category changes with the application of the kinetic estimating equation developed by Chen. We evaluated whether kinetic estimation of renal function would change medication dosing categories (≥60, 30–59, 15–29, and <15mL/min) compared with the use of CrCl or CKD-EPI eGFR.

**Results:**

The use of kinetic CrCl and CKD-EPI eGFR resulted in a large enough change in estimated renal function to require medication dosing recategorization in 19.3% [95 CI 16.8%–21.9%] and 23.4% [95% CI 20.7%–26.1%] of participants, respectively. As expected, recategorization occurred more frequently in those with AKI. When we examined individual days for those with AKI, dosing discordance was observed in 8.5% of total days using the CG CrCl and 10.2% of total days using the CKD-EPI equation compared with the kinetic counterparts.

**Conclusion:**

In a critically ill population, use of kinetic estimates of renal function impacted medication dosing in a substantial proportion of AKI participants. Use of kinetic estimates in clinical practice should lower the incidence of medication toxicity as well as avoid subtherapeutic dosing during renal recovery.

## Introduction

Acute kidney injury (AKI) is a common occurrence in hospitalized patients [1,2] and has been associated with increased mortality and longer length of stay [3]. Although newer AKI definitions incorporate urine output, AKI has traditionally been defined based on changes in serum creatinine (SCr). During AKI, when SCr is fluctuating, standard estimates of creatinine clearance (CrCl) or estimated glomerular filtration rate (eGFR) are not valid, since these equations assume SCr is at steady state. Furthermore, changes in SCr may lag behind actual changes in renal function and may take several days to reach steady state during the development of and recovery from AKI. Thus, estimated CrCl or eGFR may be higher than true GFR while AKI is developing leading to medication overdosing and lower than true GFR during recovery, leading to medication underdosing.

Equations [4–8] that incorporate the rate of SCr change have been proposed to estimate instantaneous GFR during AKI. However, they have not been widely used. In 2013, Chen [9] developed a simpler algebraic kinetic eGFR (keGFR) equation. In several small cohorts, this equation has been shown to predict renal-centered outcomes, including delayed graft function, dialysis-requiring AKI, and renal recovery better than or as well as eGFR or novel biomarkers [10–15]. As a result of these studies, keGFR is included in the Intensive Care Medicine Agenda on AKI [16] and Acute Disease Quality Initiative 16 Workgroup [17] as a tool in need of further research [18]. However, it is not necessarily surprising that keGFR is superior to standard estimates of renal function in predicting renal-centered outcomes since by definition, the former incorporates change in SCr, whereas the latter does not.

Kinetic eGFR may have significant impact for medication dosing during the development of and recovery from AKI. To date, two studies [19,20] have investigated the use of keGFR in medication dosing, but neither has quantified the magnitude of changes that would occur if keGFR was substituted for traditional methods in medication dosing. Dose adjustment based on renal function has historically used Cockcroft-Gault (CG) CrCl, since this is what has been recommended by the Food and Drug Administration for drug dosing categories during the process of drug approval. However, most electronic health records report the Chronic Kidney Disease-Epidemiology Collaboration (CKD-EPI) eGFR [21] along with SCr. In fact, the FDA 2010 draft guidance has been updated to include eGFR [22] in addition to CrCl to define renal impairment stages and prepare pharmacokinetic results [23].

We hypothesized that the use of kinetic estimates of renal function could impact drug dosing in a substantial number of critically ill patients with fluctuating renal function. The objective of the current study. was to examine the frequency with which drug dosing changes would occur. To test this hypothesis, we conducted a simulation using data from a large, well characterized, critically ill population with the acute respiratory distress syndrome (ARDS), since this population is known to have a high incidence of AKI [24].

## Materials and methods

### Data source

We used data collected from participants in the Acute Respiratory Distress Syndrome (ARDS) Network Fluid and Catheter Treatment Trial (FACTT) to quantify medication dosing changes that would occur with the application of keGFR in a critically ill population [25,26]. This data is publicly available through the National Heart, Lung and Blood Institute via BioLINCC (https://biolincc.nhlbi.nih.gov/home/). FACTT was a factorial, randomized clinical trial that assigned 1000 participants with acute lung injury to either pulmonary artery catheter versus central venous catheter as well as a fluid liberal versus fluid conservative management strategy. The trial collected daily SCr closest to 8am and daily maximum values for the first 7 days post enrollment. We limited the evaluation of kinetic estimates to 8am SCr values. If the 8am SCr value was missing (9%), the daily maximum SCr value was used. SCr was censored once dialysis was initiated. Participants with at least 2 SCr values remaining after censoring were included in the study.

### AKI definition

AKI was defined by the KDIGO consensus definition as an absolute increase in 0.3mg/dL over a 48-hour window or a relative increase in serum creatinine of 50% from baseline or need for dialysis within 7 days of enrollment. Baseline SCr was defined as the value closest to the time preceding study randomization.

### Analysis of renal function and drug dosing categories

**Fig 1:**
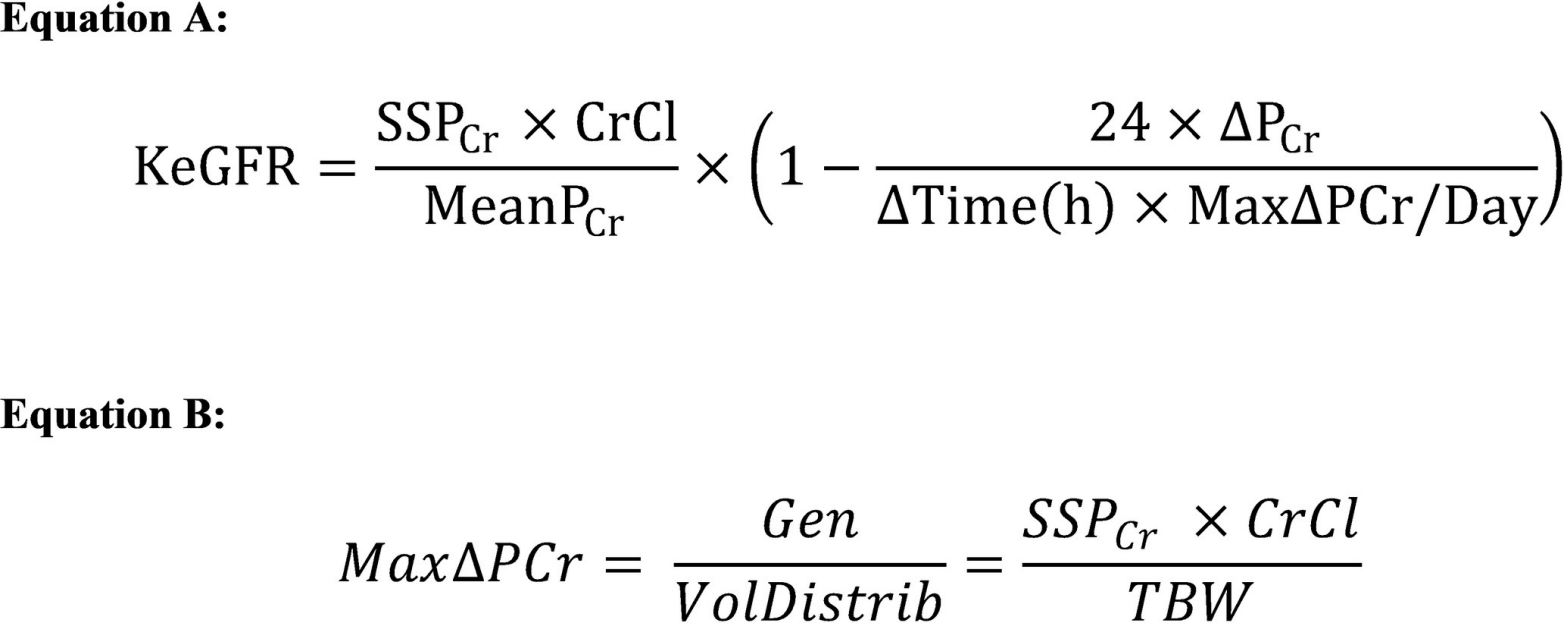
Kinetic eGFR equation as formulated by Chen et al. (Equation A) SSP_Cr_ denotes steady state plasma creatinine (in this analysis, serum creatinine at enrollment. CrCl is the estimated glomerular filtration rate calculated using the Cockcroft-Gault or CKD-EPI (unadjusted for body surface area) estimating equation with the SSP_Cr_. Mean PCr is the mean of SCr from that day and SCr from 24 hours prior. ΔPCr denotes the difference between the SCr from that day and SCr from 24 hours prior. Max ΔPCr indicates maximum change in creatinine per day as estimated by equation B. (Equation B) Max ΔPCr is calculated from the rate of creatinine generation divided by the volume of distribution. The total body water was defined as 0.6*baseline weight in kilograms.

Daily renal function was initially determined using the CG CrCl [27] or the CKD-EPI eGFR [21] equations. The CKD-EPI eGFRs were unadjusted for body surface area using the Mosteller formula- taking the square root of (height in cm *weight in kg/3600) and dividing the value by 1.73m^2^ [28,29]. These were compared to kinetic versions of these estimates calculated using the formula developed by Chen (Fig 1: Equation A) [9]. The maximum change in plasma creatinine was based upon the rate of creatinine generation divided by the volume of distribution (Fig 1: Equation B). Total body water (TBW), used to determine the volume of distribution, was defined as 0.6*baseline weight in kilograms (kg).

Equation A:

$$\text{KeGFR}=\frac{{\text{SSP}}_{\text{Cr}}\times \text{CrCl}}{{\text{MeanP}}_{\text{Cr}}}\times \left(1-\frac{24\times \Delta {\text{P}}_{\text{Cr}}}{\Delta \text{Time}\left(\text{h}\right)\times \text{Max}\Delta \text{PCr}/\text{Day}}\right)$$

Equation B:

$$Max\Delta PCr=\frac{Gen}{VolDistrib}=\frac{SSP_{Cr}\times CrCl}{TBW}$$

We categorized the standard and kinetic estimates of kidney function as recommended by the *FDA Guidance to Industry* (> = 60, 30–59, 15–29 or < 15mL/min or mL/min/1.73m^2^)[23]. The proportion of concordant and discordant category assignments between the standard versus kinetic measurements were calculated for the participants and for individual study days. We considered participants to be concordant if the standard and kinetic CrCl or eGFR categories were the same for all 7 days post-enrollment. Participants were considered discordant if the standard and kinetic CrCl or eGFR categories were not the same on at least one of those 7 days. For participants with discordant medication dosing categories, the initial change in category was used to quantify the impact of the use of kinetic eGFR or CrCl equation. 95% confidence intervals for the percentages of concordance and discordance were calculated using binomial distributions. A sensitivity analysis was also conducted where those whose estimates (standard versus kinetic) were less than 5mL/min or 5mL/min/1.73m^2^ apart were counted among the proportion of those who did not require reclassification.

### Fluid adjusted analyses

Because the FACTT trial included a fluid management strategy, and fluid overload may affect ascertainment of serum creatinine and AKI [24], a secondary analysis was completed using fluid-adjusted creatinine using the method described by Macedo et al [30]. For each study day, the cumulative on-study fluid balance was calculated using the 24 hour fluid intake and output where Adjust Cr = SCr *[1+ on study cumulative net fluid balance/TBW]. We examined the proportion of subjects requiring drug dosing reclassification in 4 groups: those who did not have AKI before or after adjustment for fluid balance, those with AKI only after adjustment for fluid balance, those who had AKI before but not after adjustment for fluid balance, and those with AKI both before and after adjustment for fluid balance, as previously described.

### Statistical analyses

Differences in means were compared using analysis of variance (ANOVA) while rank differences were compared using the Kruskal-Wallis test. Differences in proportions were conducted using Pearson’s chi squared test. Data analysis was conducted using Stata 15.1 (StataCorp, College Station, TX). P values < 0.05 were considered statistically significant.

## Results

A total of 54 FACTT participants were excluded as a result of dialysis requirement or death within day 1 of recruitment [N = 28] or missing data that did not allow for kinetic estimates [N = 26]. The final population was composed of 946 participants, with baseline characteristics as shown in Table 1. 496 (52%) developed AKI within 7 days of enrollment. An additional 110 participants developed AKI later in the study but were not considered to have AKI for this analysis. Age, sex, and weight were similar among those with and without AKI. However, there were notably more African Americans who had AKI. Patients who developed AKI were also more likely to have diabetes, hypertension, and have higher creatinine at baseline. At the time of recruitment, they were also more likely to have greater severity of illness, with higher rates of vasopressor use and higher acute physiology and chronic health evaluation III (APACHE III) scores. In the primary analysis (where there was no adjustment for differences in fluid balance between the treatment arms), participants with AKI were less likely than those without AKI to be have been randomized to the fluid liberal arm (45 vs 54%, p<0.01), similar to prior work [24,31]. Those with AKI had fewer ventilator free days (median 10 vs 21, p < 0.01) and higher 60-day mortality (16% vs 34%, p < 0.01).

**Table 1:** Baseline characteristics of all FACTT subjects included in the analysis of kinetic GFR, and then divided by the presence and absence of AKI.

	ALL subjects	No AKI	AKI	P value
Number of subjects, n	946	450	496	
**Demographics**				
Age (years)	50 ± 16	49 ± 16	50 ± 16	0.30
Female, n (%)	437 (46%)	216 (48%)	221 (45%)	0.29
African American, n (%)	200 (21%)	76 (17%)	124 (25%)	< 0.01
Weight (kg)	81 ± 23	80 ± 22	82 ± 24	0.32
Fluid Liberal Arm, n (%)	470 (50%)	246 (54%)	228 (45%)	<0.01
**Comorbidities**				
Diabetes, n (%)	162 (18%)	62 (14%)	100 (21%)	0.02
HTN, n (%)	226 (29%)	90 (24%)	136 (34%)	<0.01
**Renal Function at Time of Study Enrollment**				
Creatinine (mg/dL)	1.0 [0.7–1.5]	0.9 [0.7–1.2]	1.2 [0.8–1.7]	< 0.01
CG CrCl, ml/min	92 [58, 134]	104 [68–140]	81 [50–126]	< 0.01
CKD-EPI eGFR, ml/min/1.73m^2^	90 [55–119]	100 [69–123]	79 [47–115]	< 0.01
**Severity of Illness**				
On vasopressors at time of enrollment, n (%)	396 (42%)	173 (38%)	223 (45%)	0.04
APACHE III Score	92 ± 30	86± 28	98 ± 30	< 0.01
**Outcomes**				
Ventilator Free Days	18 [0–23]	21 [10–25]	10 [0–22]	< 0.01
Dialyzed within 60 days, n (%)	91 (10%)	4 (1%)	87 (18%)	< 0.01
Mortality within 60 days, n (%)	240 (25%)	70 (16%)	170 (34%)	< 0.01

Data is displayed as mean ± SD, median [IQR] and n (%).

**Fig 2:**
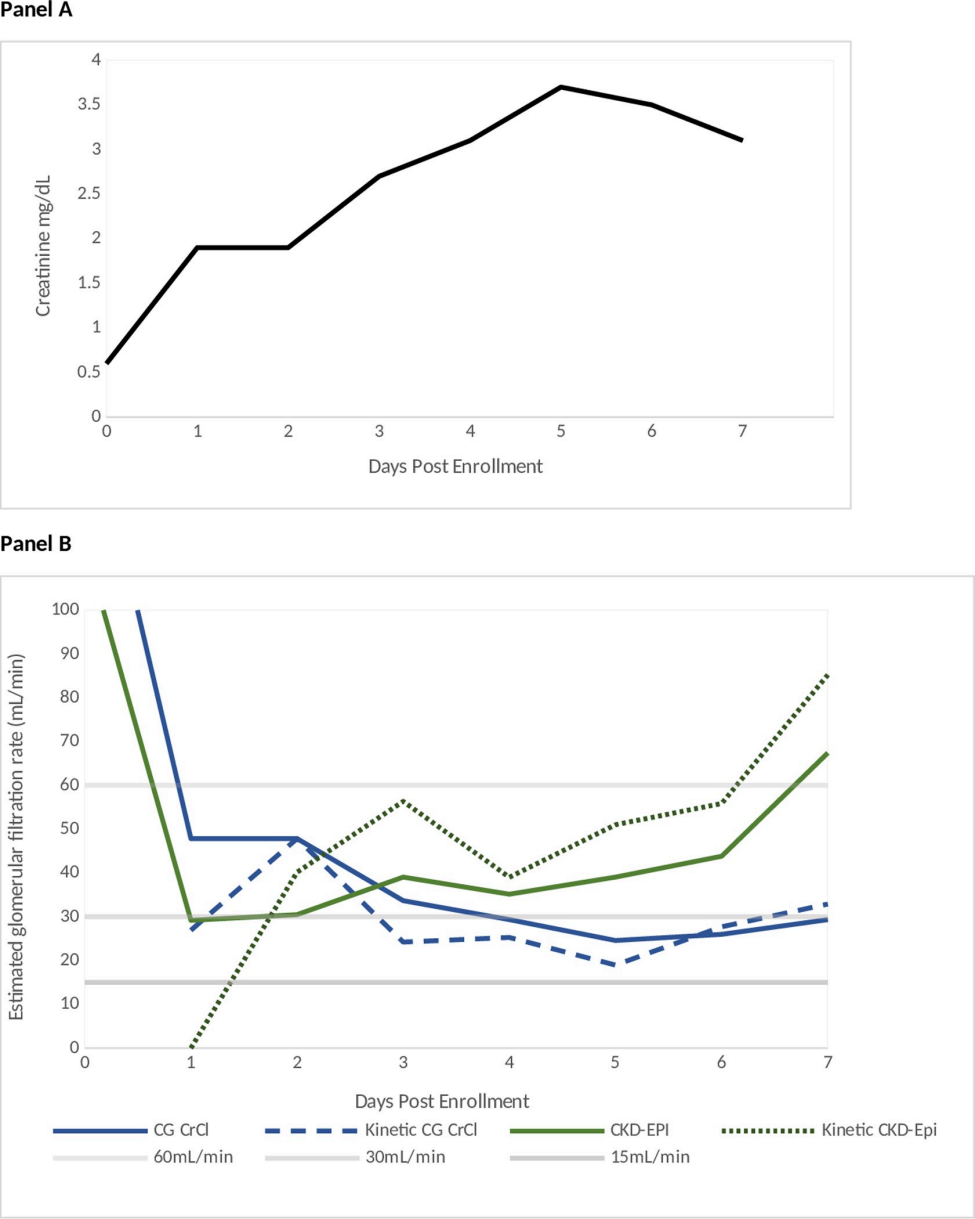
Sample application of kinetic estimate. Panel A illustrates the creatinine trajectory of one participant with AKI within the first 7 study days; Panel B illustrates the corresponding calculated CG CrCl and CKD-EPI eGFRs and their corresponding kinetic estimates. Horizontal lines in the figure demarcate FDA dosing categories (e.g., 15, 30, and 60mL/min). After the rapid creatinine rise during the development of severe AKI, the kinetic CG CrCl fell to within the 15–30mL/min dosing category on Day 1, while the CG CrCl dosing category was 30–60mL/min. This subject would be considered to have discordant drug dosing categories on this day. Conversely, on Day 7, the kinetic estimate is higher than the CG CrCl because of the lag in SCr decline during renal recovery. Using the CKD-EPI equation, discordant drug dosing categories only occurred on Day 1. On other study days, drug dosing categories were concordant.

We next compared drug dosing CrCl or eGFR categories (≥60, 30–59, 15–29, and <15mL/min) using standard and kinetic estimates. Fig 2 illustrates CrCl over time in a subject who had worsening AKI followed by renal recovery. In this subject, kinetic estimates were lower than the CrCl in the setting of AKI and worsening renal function, whereas the kinetic estimates were higher than the CrCl during recovery.

When kinetic estimates were compared to standard estimates, 19.3% [95% CI 16.8%–21.9%] of participants and 23.4% [95% CI 20.7%–26.1%] required any change in dosing category on at least one study day using the CG CrCl and unadjusted for BSA CKD-EPI equations, respectively (Table 2). Those with AKI were more likely to have discordant standard and kinetic dosing categories. For example, when CG CrCl was used, 33.5% [95% CI 29.3%–37.6%] of those with AKI versus 3.8% [95% CI 2.0%-5.5%] of those without AKI required any change in dosing category using the kinetic estimate. Those who were not classified as having AKI mostly required dosing adjustment during falls in serum Cr which likely represent recovery from AKI. In a sensitivity analysis, we removed small changes (< 5mL/min between kinetic and nonkinetic estimates) from the recategorization analysis. For example, in the sensitivity analysis, if the CrCl was 32mL/min and the kinetic CrCl was 28mL/min, the participant was not considered to have a change in medication dosing category for that day. There was a smaller proportion of individuals with discordant dosing categories (13.6% for CG Cl, [95% CI 11.4%-15.8%]; 16.0% [95% CI 13.7–18.4%] for CKD-EPI eGFR, S1 Table) but the proportion was still substantial.

**Table 2:** Dosing recategorization using kinetic estimates of CrCl and eGFR. The percentage of patients who required recategorization with use of kinetic estimates of each formula is shown, stratified by AKI status. The number of drug dosing categories crossed (>60, 30–60, 15–29, < 15ml/min) refers to the initial study day that redosing was required.

ALL (n = 946)	No change % (n) [95% CI]	±1 category % (n) [95% CI]	±2 categories % (n) [95% CI]
Cockcroft-Gault CrCl	**80.7%** (763) [78.1%, 83.1%]	**18.3**% (173) [15.8%, 20.8%]	**1.0%** (10) [0.4%-1.7%]
CKD-EPI	**76.6%** (725) [73.9%-79.3%]	**22.6%** (214) [20.0%, 25.3%]	**0.7%** (7) [0.1%-1.3%]
**No AKI (n = 450)**	**No change**	**±1 category**	**±2 categories**
Cockcroft-Gault CrCl	**96.2%** (433) [94.5%-98.0%]	**3.8%** (17) [2.0%-5.5%]	**0%** (0) —
CKD-EPI	**92.7%** (417) [90.3%-95.1%]	**7.3%** (33) [4.9%-9.7%]	**0%** (0) —
**AKI (n = 496)**	**No change**	**±1 category**	**±2 categories**
Cockcroft-Gault CrCl	**66.5%** (330) [62.4–70.7%]	**31.5%** (156) [27.3%-35.5%]	**2.0%** (10) [0.8%-3.3%]
CKD-EPI	**62.1%** (308) [57.8%-66.4%]	**36.5%** (181) [32.3%-40.7%]	**1.4%** (7) [0.3%-2.4%]

For a given participant, the use of kinetic estimates may only affect drug dosing practices on a subset of study days. As illustrated by data from a study participant with AKI in Fig 2, kinetic estimates would have changed daily dosing practices on 3 of 7 study days (Day 1, 3, and 7). When we examined individual days for the entire study population (Table 3), dosing discordance was observed in 4.6% of total days using the CG CrCl and 6.0% of total days using the CKD-EPI equation (S2 Table). Irrespective of AKI status, concordance between CG CrCl and kinetic GFR estimated drug categories decreased with lower GFR. In those with AKI, the drug dosing discordance increased to 8.5% of total days using the CG CrCl equation and 10.2% of total days using the CKD-EPI equation. Discordance occurred during both the establishment of AKI and recovery. The discrepancy occurred most frequently immediately post study enrollment and declined over time (S3 Table). The decline in discordance over time was more prominent when the CG CrCl was applied compared to the CKD-EPI equation.

**Table 3:** Dosing recategorization by study days. Comparison of dosing categories for all study days using the kinetic versus standard Cockcroft Gault CrCl in all subjects and then divided by AKI status. Subjects along the diagonal have concordant drug dosing using the two estimates, whereas those in the off-diagonal cells have discordant drug dosing using the two estimates. The majority of drug redosing occurs in subjects who experienced AKI.

ALL Subjects	Kinetic CG CrCl Categories (4.6% Recategorized)				
**CG CrCl Categories**	**> = 60mL/min**	**30–59 mL/min**	**15–29mL/min**	**< 15mL/min**	**Total**
**> = 60 mL/min**	4481	39	0	0	4520
**30–59 mL/min**	28	848	77	10	963
**15–29 mL/min**	0	30	222	82	334
**< 15 mL/min**	0	0	4	49	53
**Total**	4509	917	303	141	**5870 days**
**No AKI**	**Kinetic CG CrCl Categories (0.6% Recategorized)**				
**CG CrCl Categories**	**> = 60mL/min**	**30–59 mL/min**	**15–29mL/min**	**< 15mL/min**	**Total**
**> = 60 mL/min**	2597	2	0	0	2599
**30–59 mL/min**	10	272	1	0	283
**15–29 mL/min**	0	4	24	0	28
**< 15 mL/min**	0	0	0	1	1
**Total**	2607	278	25	1	**2911 days**
**AKI**	**Kinetic CG CrCl Categories (8.5% Recategorized)**				
**CG CrCl Categories**	**> = 60mL/min**	**30–59 mL/min**	**15–29mL/min**	**< 15mL/min**	**Total**
**> = 60 mL/min**	1884	37	0	0	1921
**30–59 mL/min**	18	576	76	10	680
**15–29 mL/min**	0	26	198	82	306
**< 15 mL/min**	0	0	4	48	52
**Total**	1902	639	278	140	**2959 days**

As a final sensitivity analysis, because the FACTT trial involved a specific fluid management strategy, the analysis was repeated using fluid corrected SCr as described in Macedo et al (S4 Table) [30]. 3 subjects were excluded because they had only 1 day of fluid data. With fluid correction, the proportion of subjects requiring drug dosing recategorization increased overall (from 19.4% to 23.4% using the CG CrCl equation and from 23.4% to 25.2% using the CKD-EPI equation). Recategorization occurred least in those who did not meet the AKI definition using either non-fluid corrected or fluid corrected creatinine values (3.2% using the CG CrCl equation and 3.9% using the CKD-EPI equation) and most in those who met AKI definitions using both non-fluid corrected and fluid corrected group (44.0% using the CG CrCl equation and 44.2% using the CKD-EPI equation).

## Discussion

At present, there is no standardized way to dose medications in AKI given fluctuating kidney function and filtration markers such as SCr. This is problematic during the development of AKI, when CrCl or eGFR will overestimate renal function and may lead to drug accumulation and potential toxicity as well as during renal recovery, when CrCl or eGFR will underestimate renal function and therapeutic drug levels may not be attained. There has been interest in the use of kinetic equations to refine estimates of renal function. Here, we show in a critically ill population of patients with ARDS that the use of the Chen kinetic estimating equation might affect drug dosing in a quarter of critically ill patients. Amongst those with AKI, drug dosing would be impacted in 33.5% and 37.9% of patients using the CrCl equation and CKD-EPI equation, respectively.

Furthermore, we found that the kinetic equation impacts drug dosing the most at lower eGFRs. This may be because at lower estimates of renal function, a 50mL/min adjustment (e.g. from 60 to 10mL/min) by the kinetic equation results in a change of two dosing categories. In contrast, at higher levels of renal function, similar adjustments (e.g. from 120 to 70mL/min) would not require any drug redosing. The impact of kinetic equations is also most prominent in the first few days of hospitalization and wanes over time, especially when kinetic CrCl is applied rather than the kinetic CKD-EPI equation. We suspect that the differential impact of the two equations over time is secondary to lower serum creatinine values observed during renal recovery later in the hospitalization. Traditionally, the CG CrCl equation results in higher estimates of renal function compared to the CKD-EPI equation at low values of serum creatinine, and, as described above, at higher levels of estimated renal function, kinetic adjustments are less likely to result in changes in drug dosing categories.

Over the last few years, Chen’s kinetic equation has been increasingly used and validated in various settings. In the intensive care setting, keGFR performed better than several novel biomarkers in predicting renal recovery and major adverse kidney events [11]. KeGFR has been also tested in patients undergoing cardiac surgery for predicting AKI and operative mortality[14]. The equation has also been implemented in the renal transplant population to improve early AKI detection in living donors [32], to predict delayed graft function in renal transplant recipients [10,13] and as a secondary endpoint for a randomized clinical trial where those randomized to balanced salt solutions (Plasma-lyte) versus normal saline trended towards better keGFR [33]. Together, these studies show that keGFR improves prediction and understanding of renal function in AKI when the serum creatinine is rapidly fluctuating. However, there are limited studies evaluating keGFR for the purposes of drug dosing[19,20]. Harada [19] et al. compared measured vancomycin concentrations with predicted vancomycin concentrations derived using varying estimates of renal function, but the predicted concentration [34] was derived from CG CrCl, which will bias the results in favor of the CG equation. Pharmacist-led medication dosing tends to use CG CrCl because the previous US Food and Drug Administration guidance on pharmacokinetic categories is based on CrCl. In contrast, nephrologists typically use eGFR estimates for medication dosing in CKD, which may lead to discrepant dosing recommendations [35,36].

In AKI, steady state estimating equations perform poorly. In a study conducted by Bragadottir et al. [37], the within group-error was 68.7%, 67.7% and 68.0% for MDRD eGFR, CKD-EPI eGFR and CG CrCl respectively when compared to the measured GFR (measured by ^51^Cr-EDTA) in early AKI. Equations that account for SCr kinetics may improve the evaluation of renal function in AKI. The Jelliffe formula, one of the first kinetic GFR equations, has been shown to be superior to estimating equations in small prior studies [6,38]. In AKI, eGFR by the CG CrCl, MDRD eGFR and Jelliffe formulas overestimated urinary creatinine clearance 80%, 33% and 10% of the time respectively [38]. Although the Chen formula was not tested, these results imply that kinetic approaches may improve the evaluation of kidney function in AKI. Compared to earlier formulas, the Chen formula provides algebraic simplicity that allows for easier implementation into clinical practice.

Our study sought to determine the impact of keGFR on drug dosing if keGFR was widely applied into the electronic health record, eliminating the need for labor intensive timed urine clearance measurements. Although therapeutic drug level monitoring can be used for certain medications (e.g., vancomycin), there are relatively few medications where real-time monitoring is feasible. Commonly used, important medications in critically ill patients often require redosing based on GFR category. For example, the therapeutic dose range for cefepime varies from 2 g every 8 hours in the setting of normal renal function to 1 g intravenous every 24 hours in the setting of GFR < 10 mL/min for pseudomonal infections. Cefepime accumulation can lead to encephalopathy and coma, yet underdosing can lead to inadequate treatment of infections. Incorporation of keGFR into the electronic health record may help critical care providers easily adjust cefepime dosing daily to avoid toxicity in the setting of worsening AKI and, just as importantly, avoid underdosing during renal recovery. Future approaches may also include the use of novel filtration biomarkers such as cystatin C and real time GFR measurement, which are likely to be soon available to evaluate drug clearance in AKI. However, it is unlikely that real time GFR measurement will be implementable in all patients across all settings. Therefore, further studies are necessary to compare keGFR with measured GFR to allow for a cost-effective and efficient way for estimating GFR in AKI.

The strengths of this study include the use of a diverse, large critically ill population with daily creatinine data. We selected drug dosing categories that are large and clinically meaningful. Therefore, the results should be relatively robust to small changes in eGFR. Furthermore, there is limited missing data. In cases where patients were excluded due to kinetic estimates not available secondary to death/early dialysis, it is presumed that if kinetic GFR was applied at later dates, the proportion of participants who need dosing adjustment with application of keGFR would increase. Thus, this study provides a relatively conservative estimate of those who would be affected.

This study has some limitations. First, while we quantified the proportion of participants and days that required a change in medication dosing, we did not have drug toxicity or levels available; in fact, drug levels are rarely available to guide dosing. As such, we cannot identify whether redosing medications using these kinetic formulas would directly impact participant outcomes. Higher mortality was observed in those who would have required medication redosing, but this is likely due to the severity of illness associated with AKI rather than medication toxicity or subtherapeutic medications.

## Conclusions

In this large critically ill population with ARDS and an overall AKI rate of 52%, the use of kinetic estimates of renal function would likely impact drug dosing in a quarter of all subjects during the first week of admission. Most of this drug redosing would occur in participants with AKI, and in this analysis, approximately 8–10% of study days among those with AKI would be impacted. Thus, the magnitude of drug redosing that might occur in critically ill patients with the use of keGFR warrants additional studies to further test the clinical utility of keGFR.

## Supporting information

S1 Table Sensitivity analysis eliminating changes in dosing category due to differences of less than 5 mL/min between standard and kinetic estimates. (DOCX)

S2 Table Dosing category changes using standard and kinetic CKD-EPI eGFR. (DOCX)

S3 Table Discordant dosing categories in both equations across study days. (DOCX)

S4 Table Fluid adjusted analysis of standard versus kinetic renal function. (DOCX)
