# Supplementary material for: The value of kinetic glomerular filtration rate estimation on medication dosing in acute kidney injury
Source: PLoS One. 2019 Nov 26;14(11):e0225601. doi: 10.1371/journal.pone.0225601 (PMC6879155; doi:10.1371/journal.pone.0225601)
Supplement: S1 Table — (DOCX) [file pone.0225601.s001.docx]

**S1 Table: Sensitivity analysis eliminating changes in dosing category due to differences of less than 5 mL/min between standard and kinetic estimates** For this analysis, to exclude those where small changes in CrCl or eGFR estimates accounted for a change in drug dosing category, if the difference between the standard and kinetic estimates was <5mL/min, the drug dosing categorization was considered unchanged.

| **ALL (n=946)** | **No change % (n)**  **[ 95% CI]** | **±1 category % (n)**  **[ 95% CI]** | **±2 categories % (n)**  **[ 95% CI]** |
| --- | --- | --- | --- |
| Cockcroft-Gault CrCl | **86.4%** (817)  [84.2%-88.6%] | **12.6%** (119)  [10.5%-14.7%] | **1.0%** (10)  [0.4%-1.7%] |
| CKD-EPI | **83.9%** (794)  [81.6%-86.3%] | **15.3%** (145)  [13.0%-17.6%] | **0.7%** (7)  [0.2%-1.3%] |
| **No AKI (n=463)** | **No change** | **±1 category** | **±2 categories** |
| Cockcroft-Gault CrCl | **98.0%** (441)  [96.7%-99.3%] | **2.0%** (9)  [0.7%-3.3%] | **0.0%** (0)  -- |
| CKD-EPI | **96.4%** (434)  [94.7%-98.2%] | **3.6%** (16)  [1.8%-5.3%] | **0.0%** (0)  -- |
| **AKI (n=491)** | **No change** | **±1 category** | **±2 categories** |
| Cockcroft-Gault CrCl | **75.8%** (376)  [72.0%-79.6%] | **22.2%** (110)  [22.2 %-25.8%] | **2.0%** (10)  [0.8%-3.3%] |
| CKD-EPI | **72.6%** (360)  [68.7%-76.5%] | **26.0%** (129)  [22.1%-29.9%] | **1.4%** (7)  [0.4%-2.4%] |
