# Supplementary material for: The value of kinetic glomerular filtration rate estimation on medication dosing in acute kidney injury
Source: PLoS One. 2019 Nov 26;14(11):e0225601. doi: 10.1371/journal.pone.0225601 (PMC6879155; doi:10.1371/journal.pone.0225601)
Supplement: S2 Table — (DOCX) [file pone.0225601.s002.docx]

**S2 Table: Dosing categories changes using standard and kinetic CKD-EPI eGFR.** Comparison of dosing categories for all study days using the standard versus kinetic CKD-EPI eGFR in all subjects and then stratified by AKI status.

| **ALL Subjects** | **Kinetic CKD-EPI Categories (6.0% recategorized)** | | | | |
| --- | --- | --- | --- | --- | --- |
| **CKD-EPI Categories (mL/min/1.73m^2^)** | **>=60** | **30-59** | **15-29** | **< 15** | **Total** |
| **>=60** | 4378 | 85 | 0 | 0 | 4463 |
| **30-59** | 47 | 819 | 54 | 7 | 927 |
| **15-29** | 0 | 50 | 230 | 97 | 377 |
| **< 15** | 0 | 0 | 12 | 91 | 103 |
| **Total** | 4425 | 954 | 296 | 195 | **5870 days** |
| **No AKI** | **Kinetic CKD-EPI Categories (1.7% recategorized)** | | | | |
| **CKD-EPI Categories (mL/min/1.73m^2^)** | **>=60** | **30-59** | **15-29** | **< 15** | **Total** |
| **>=60** | 2576 | 31 | 0 | 0 | 2607 |
| **30-59** | 8 | 264 | 0 | 0 | 272 |
| **15-29** | 0 | 10 | 21 | 0 | 31 |
| **< 15** | 0 | 0 | 0 | 1 | 1 |
| **Total** | 2584 | 305 | 21 | 1 | **2911 days** |
| **AKI** | **Kinetic CKD-EPI Categories (10.2% recategorized)** | | | | |
| **CKD-EPI Categories (mL/min/1.73m^2^)** | **>=60** | **30-59** | **15-29** | **< 15** | **Total** |
| **>=60** | 1802 | 54 | 0 | 0 | 1856 |
| **30-59** | 39 | 555 | 54 | 7 | 655 |
| **15-29** | 0 | 40 | 209 | 97 | 346 |
| **< 15** | 0 | 0 | 12 | 90 | 102 |
| **Total** | 1841 | 659 | 275 | 194 | **2959 days** |
