# Supplementary material for: The value of kinetic glomerular filtration rate estimation on medication dosing in acute kidney injury
Source: PLoS One. 2019 Nov 26;14(11):e0225601. doi: 10.1371/journal.pone.0225601 (PMC6879155; doi:10.1371/journal.pone.0225601)
Supplement: S3 Table — (DOCX) [file pone.0225601.s003.docx]

**S3 Table: Discordant dosing categories by study day**. Discordant drug categories are presented by study day, using CrCl and CKD-EPI eGFR.

|  | **Discordant drug categories using CrCl vs kinetic CrCl** | | **Discordant drug categories using CKD-EPI vs kinetic CKD-EPI** | | **Total** |
| --- | --- | --- | --- | --- | --- |
|  | **n** | **%**  **(95% CI)** | **n** | **%**  **(95% CI)** |  |
| Study day 1 | 68 | 7.2%  (5.6%-8.9%) | 80 | 8.5%  (6.7-10.3%) | 942 |
| Study day 2 | 49 | 5.5%  (4.0%-7.0%) | 58 | 6.5%  (4.9%-8.1%) | 895 |
| Study day 3 | 48 | 5.5%  (4.0%-7.0%) | 45 | 5.1%  (3.7%-6.6%) | 874 |
| Study day 4 | 33 | 3.9%  (2.6%-5.2%) | 37 | 4.4%  (3.0%-5.9%) | 841 |
| Study day 5 | 27 | 3.3%  (2.1%-4.6%) | 45 | 5.6%  (4.0%-7.2%) | 807 |
| Study day 6 | 23 | 3.0%  (1.8%-4.2%) | 40 | 5.3%  (3.7%-6.8%) | 761 |
| Study day 7 | 22 | 2.9%  (1.7%-4.1%) | 47 | 6.3%  (4.5%-8.0%) | 750 |
