# Supplementary material for: The value of kinetic glomerular filtration rate estimation on medication dosing in acute kidney injury
Source: PLoS One. 2019 Nov 26;14(11):e0225601. doi: 10.1371/journal.pone.0225601 (PMC6879155; doi:10.1371/journal.pone.0225601)
Supplement: S4 Table — (DOCX) [file pone.0225601.s004.docx]

**S4 Table: Fluid adjusted analysis of standard versus kinetic renal function.** Percentage of patients who required recategorization using fluid adjusted creatinine to define standard and kinetic renal function. The data is stratified into 4 groups based on AKI status with or without fluid correction. The number of drug dosing categories crossed (>60, 30-60, 15-29, < 15ml/min) refers to the initial study day that redosing was required.

| **ALL (n =943)*** | **No change** | **±1 category** | **±2 categories** |
| --- | --- | --- | --- |
| Cockcroft-Gault CrCl | 723 (76.7%) | 208 (22.1%) | 12 (1.3%) |
| CKD-EPI | 706 (74.9%) | 228 (24.2%) | 9 (1.0%) |
| **No AKI in Non-Fluid and Fluid Corrected Group (n=312)** | **No change** | **±1 category** | **±2 categories** |
| Cockcroft-Gault CrCl | 302 (96.8%) | 10 (3.2%) | 0 (0.0%) |
| CKD-EPI | 300 (96.2%) | 12 (3.9%) | 0 (0.0%) |
| **No AKI in Non-Fluid Corrected and AKI in Fluid Corrected Group (n=138)** | **No change** | **±1 category** | **±2 categories** |
| Cockcroft-Gault CrCl | 123 (89.1%) | 15 (10.9 %) | 0 (0.0%) |
| CKD-EPI | 109 (79.0%) | 29 (21.0%) | 0 (0.0%) |
| **AKI in Non-Fluid Corrected and No AKI in Fluid Corrected Group (n=56)** | **No change** | **±1 category** | **±2 categories** |
| Cockcroft-Gault CrCl | 53 (94.6%) | 3 (5.4%) | 0 (0.0%) |
| CKD-EPI | 53 (94.6%) | 3 (5.4%) | 0 (0.0%) |
| **AKI in Non-Fluid and Fluid Corrected Group**  **(n=437)** | **No change** | **±1 category** | **±2 categories** |
| Cockcroft-Gault CrCl | 245 (56.1%) | 180 (41.2 %) | 12 (2.8%) |
| CKD-EPI | 244 (55.8%) | 184 (42.1%) | 9 (2.1%) |

*3 patients only had 1 day of fluid data, corrected creatinine was missing for day 2 so the standard and kinetic fluid corrected renal function estimate could not be compared.
